# Welfare receipt and the risk of vitamin D deficiency in Japanese patients on maintenance hemodialysis: a cross-sectional, retrospective study

**DOI:** 10.1186/s41100-021-00364-6

**Published:** 2021-08-26

**Authors:** Keisuke Yoshida, Tomoki Yonaha, Masayuki Yamanouchi, Hirofumi Sumi, Yasuhiro Taki, Yuhei Otobe, Minoru Miyashita, Rina Hachisuka, Wei Han, Yugo Shibagaki, Naoto Tominaga

**Affiliations:** 1 Division of Nephrology and Hypertension, Kawasaki Municipal Tama Hospital, 1-30-37, Shukugawara, Tama-ku, Kawasaki, Kanagawa 214-8525 Japan; 2 Division of Nephrology and Hypertension, Department of Internal Medicine, St. Marianna University School of Medicine, 2-16-1, Sugao, Miyamae-ku, Kawasaki, Kanagawa 216-8511 Japan; 3 Nephrology and Dialysis Center, Yonaha Medical Clinic, 2287-35, Arakawa, Ishigaki, Okinawa 907-0024 Japan; 4 Nephrology Center, Toranomon Hospital Kajigaya, 1-3-1, Kajigaya, Takatsu-ku, Kawasaki, Kanagawa 213-8587 Japan; 5 Department of Rehabilitation Medicine, Kawasaki Municipal Tama Hospital, 1-30-37, Shukugawara, Tama-ku, Kawasaki, Kanagawa 214-8525 Japan; 6 Department of Nutrition, Kawasaki Municipal Tama Hospital, 1-30-37, Shukugawara, Tama-ku, Kawasaki, Kanagawa 214-8525 Japan

**Keywords:** Maintenance hemodialysis, Vitamin D deficiency, Serum 25-hydroxyvitamin D concentration, Welfare receipt

## Abstract

**Background:**

Vitamin D deficiency is often observed in patients undergoing maintenance hemodialysis and is associated with significantly increased risk of overall mortality. Despite reports of poor nutrition/intake, vitamin D status among patients on maintenance hemodialysis receiving welfare remains unknown. This study investigated the vitamin D status in welfare recipients undergoing maintenance hemodialysis.

**Methods:**

This cross-sectional study investigated vitamin D status among 106 outpatients undergoing maintenance hemodialysis at two medical facilities in Japan. Patients were divided into welfare and non-welfare groups based on their status as of September 2018. Patients were divided into two categories: serum vitamin D deficiency, defined as serum 25(OH)D concentrations < 12 ng/mL, or non-deficiency. Vitamin D deficiency was used as a dependent variable, while welfare receipt was used as the main predictor variable.

**Results:**

Mean [± standard deviation] patient age, median [interquartile range] body mass index, and hemodialysis duration were 66.9 [± 10.8] years, 21.5 [19.6, 24.3] kg/m^2^, and 7.9 [2.9, 12.3] years, respectively. Among 106 patients, 45 were women (42.5%) and 16 (15.1%) were receiving welfare. The welfare group had a higher diabetes prevalence (*P* = 0.003) and significantly lower median serum 25-hydroxyvitamin D concentrations (11.5 [8.7, 14.0] vs. 14.8 [11.2, 19.9] ng/mL, *P* = 0.005). Multiple logistic regression analysis revealed that welfare receipt was a significant risk factor for vitamin D deficiency (odds ratio [95% confidence interval], 4.41 [1.08, 18.07]).

**Conclusions:**

Welfare recipients undergoing maintenance hemodialysis are at significantly increased risks of vitamin D deficiency compared with patients not receiving welfare.

## Background

Vitamin D (VD) is an essential hormone for maintaining health [1]. Although 1,25-dihydroxycholecalciferol [1,25(OH)_2_D] is the active form of VD, the most reliable marker of VD status is 25-hydroxyvitamin D [25(OH)D] [2]. Therefore, 25(OH)D is an important nutritional index that best reflects VD status in the body based on the supply obtained from the skin and food. For adults, most authors define VD deficiency as concentrations < 12 ng/mL [3].

However, in modern society, serum 25(OH)D concentrations can be low owing to imbalanced eating habits and reduced sunlight exposure duration (or excessive ultraviolet protection). Deficiency of 25(OH)D increases the risk of bone fracture due to osteoporosis and the risk of overall mortality [4]; this finding has also been observed among patients undergoing hemodialysis (HD) [5]. Insufficiency of 25(OH)D is commonly observed in patients undergoing maintenance HD, and such patients often exhibit poor outcomes, including increased mortality [6–8]. Nevertheless, some questions regarding indications for measuring serum 25(OH)D concentration in patients undergoing maintenance HD remain unanswered.

Public health efforts should focus on improving VD status not only by clarifying the relative contributions of known determinants but also by identifying new determinants, given that many patients on maintenance HD may exhibit VD deficiency. As of September 2018, the number of individuals receiving welfare, known as Public Assistance (Seikatsu Hogo) in Japan, had increased to over 2 million (1.66% of the population). Among the types of social welfare available in Japan, Public Assistance serves to provide for those who are ineligible for social security benefits [9, 10]. Nutritional imbalances and inadequate consumption of essential nutrients have been reported among welfare recipients [11]. While several studies have discussed VD status among patients undergoing HD, VD status among welfare recipients undergoing HD remains unknown.

This study aimed to compare VD status between two groups of patients undergoing HD, namely welfare and non-welfare groups, and examine whether welfare recipients undergoing maintenance HD were at risks of VD deficiency.

## Materials and methods

### Patient population and data source

Data were obtained from the pooled patient electronic health records system for Kawasaki Municipal Tama Hospital in Kanagawa and Yonaha Medical Clinic in Okinawa. We retrospectively reviewed clinical and laboratory data of outpatients who underwent maintenance HD between April 2018 and September 2018.

This study was conducted according to the Declaration of Helsinki and approved by the institutional review board (IRB) at St. Marianna University School of Medicine (IRB Approval Number: 4630). The requirement for informed consent was waived because of the retrospective nature of this study and the de-identified nature of the analyses.

### Study design and procedures

This cross-sectional study investigated whether welfare receipt (as a binary predictor variable) was a risk factor for VD deficiency, defined as serum 25(OH)D concentrations < 12 ng/mL [3] (as a binary dependent variable) among patients undergoing HD. Patients undergoing HD for < 6 months and those undergoing combined HD and peritoneal dialysis were excluded. Similarly, to exclude potential effects of other pathologies on VD status, we excluded bone metastasis and/or multiple myeloma patients. No patients at either facility had undergone small bowel resection or had been diagnosed with inflammatory bowel or granulomatous diseases.

We selected known-risk factors and those associated with low serum 25(OH)D concentration [12–21] and utilized the HD vintage, the means of 11 consecutive values for hemoglobin (Hb), serum albumin (Alb), corrected calcium (cCa), phosphorus (P), and C-reactive protein (CRP) concentrations measured in the blood twice per month, as well as the means of six consecutive values for plasma intact parathyroid hormone (iPTH) concentration measured once per month before obtaining serum 25(OH)D concentration data as of September 2018. Serum 25(OH)D concentration was measured using the chemiluminescence immunoassay method, and plasma iPTH concentration was measured using the electrochemiluminescence immunoassay method. All blood tests were performed before HD at the first HD session of the week. Patient exposures to the clinical variables of interest were defined by documenting diagnosis (i.e., diabetes), medication (i.e., immunosuppressants), and HD vintage. Body mass index (BMI) was calculated by dividing weight (kg) by height squared (m^2^).

The International Physical Activity Questionnaire (IPAQ) is widely used to assess physical activity levels in adults and adolescents [22]. The IPAQ assesses physical activity undertaken across a comprehensive set of domains, including the following: (1) leisure-time physical activity, (2) domestic and gardening (yard) activities, (3) work-related physical activity, and (4) transport-related physical activity. In this study, physical activity levels were assessed using the IPAQ-Short Form (IPAQ-SF), which includes items related to three specific types of activity undertaken in the four domains mentioned above [23]. The IPAQ-SF provides separate scores for walking, moderate-intensity activity, and vigorous-intensity activity. Total IPAQ-SF scores are obtained by summing the duration (in minutes) and frequency (in days) of activity for each of the three intensity levels. All continuous scores were expressed in metabolic equivalent (MET) × minutes/week. The following values are typically used to analyze IPAQ-SF data: walking = 3.3 METs, moderate-intensity physical activity = 4.0 METs, and vigorous-intensity physical activity = 8.0 METs. Using these values, four continuous scores are defined as follows:

$$\begin{aligned} & {\text{Walking}}\,{\text{MET}} \times {\text{minutes/week}} = 3.3 \\ & \quad \times {\text{walking}}\,{\text{minutes}} \times {\text{walking}}\,{\text{days}} \\ & {\text{Moderate MET}} \times {\text{minutes}}/{\text{week}} = {4}.0 \times {\text{moderate}} \\ & \quad - {\text{intensity activity minutes}} \\ & \quad \times {\text{moderate}} - {\text{intensity days}} \\ & {\text{Vigorous MET}} \times {\text{minutes}}/{\text{week}} = {8}.0 \times {\text{vigorous}} \\ & \quad - {\text{intensity activity minutes}} \\ & \quad \times {\text{vigorous}} - {\text{intensity days}} \\ & {\text{Total}}\,{\text{physical}}\,{\text{activity}}\,{\text{MET}} \times {\text{minutes/week}} \\ & \quad = {\text{sum}}\,{\text{of}}\,{\text{Walking}} + {\text{Moderate}} + {\text{Vigorous}}\,{\text{MET}} \\ & \quad \quad \times {\text{minutes/week}}\,{\text{scores}}. \\ \end{aligned}$$

### Statistical analyses

All data are summarized using descriptive statistics, including means, standard deviations (SD), medians, interquartile ranges (IQRs), frequencies, and percentages. For continuous variables, Student’s t tests or Mann–Whitney U-tests were used to assess the significance of differences in patient characteristics between the welfare and non-welfare groups. For discrete variables, Chi-square tests were used to compare data between the two groups. Single and multiple logistic regression analyses were used to examine whether the known risk factors and/or welfare receipt increased the risk of VD deficiency as of September 2018. A multiple linear regression analysis was performed using variables with *P* < 0.1 in the single linear regression analysis (model 1) and serum cCa and P concentrations in addition to the variables in model 1 (model 2). In the single and multiple linear regression analyses, values of BMI, HD vintage, Hb, serum CRP, and plasma iPTH concentrations were transformed to natural logarithms (ln) for their skewed distribution. Statistical analyses were performed using Stata version 14 (StataCorp LP, College Station, TX, USA). Statistical significance was defined as *P* < 0.05.

## Results

### Patient characteristics

Mean patient age and median BMI were 66.9 years and 21.5 kg/m^2^, respectively. Mean serum Alb and median serum 25(OH)D concentrations were 3.6 g/dL and 14.3 ng/mL, respectively. Among 106 included patients, 45 were women (42.5%) and 16 were welfare recipients (15.1%), as shown in Table 1.

**Table 1:** Characteristics of participants in the two groups

Parameter	Total	Welfare status		*P* value
		Non-welfare	Welfare	Non-welfare versus Welfare
*N* (%)	106 (100)	90 (84.9)	16 (15.1)	–
Age, years	66.9 ± 10.8	67.0 ± 11.0	66.3 ± 10.0	0.810
Female sex, *n* (%)	45 (42.5)	40 (44.4)	5 (31.3)	0.325
BMI, kg/m^2^	21.5 [19.6, 24.3]	21.5 [19.6, 24.3]	22.9 [19.6, 25.1]	0.589
HD vintage, year	7.9 [2.9, 12.3]	8.4 [3.0, 12.5]	6.9 [2.8, 8.8]	0.218
Diabetes, *n* (%)	38 (35.8)	27 (30.0)	11 (68.8)	0.003^*^
History of immunosuppressant use, *n* (%)	7 (6.6)	7 (7.8)	0 (0)	0.248
IPAQ-SF, METs × minutes/week	724.5 [192, 2079]	823.5 [266.0, 1891.5]	357.0 [0, 2423.0]	0.292
Hb, g/dL	11.1 [10.7, 11.7]	11.1 [10.8, 11.7]	10.8 [10.5, 11.5]	0.246
CRP, mg/dL	0.12 [0.05, 0.31]	0.10 [0.05, 0.26]	0.25 [0.06, 0.44]	0.162
Alb, g/dL	3.6 ± 0.3	3.6 ± 0.3	3.5 ± 0.3	0.370
cCa, mg/dL	9.0 ± 0.7	9.0 ± 0.7	8.8 ± 0.8	0.206
P, mg/dL	5.4 ± 1.1	5.4 ± 1.1	4.9 ± 1.0	0.103
iPTH, pg/mL	199.1 [119.0, 277.2]	202.6 [121.4, 281.0]	129.8 [111.4, 253.4]	0.370
25(OH)D, ng/mL	14.3 [11.0, 18.9]	14.8 [11.2, 19.9]	11.5 [8.7, 14.0]	0.005^*^
VD category (deficiency/non-deficiency), *n*	32/74	24/66	8/8	0.061

Abbreviations: Alb: albumin; BMI, body mass index; cCa, corrected calcium; CRP, C-reactive protein; Hb, hemoglobin; HD, hemodialysis; IPAQ-SF, International Physical Activity Questionnaire-Short Form; iPTH, intact parathyroid hormone; MET, metabolic equivalent; P, phosphorus; 25(OH)D, 25-hydroxyvitamin D; VD, vitamin D

Values are expressed as the mean ± standard deviation, the median [interquartile range] or as frequencies and percentages [*n* (%)]

^*^P < 0.01

### Comparison between the welfare and non-welfare groups

We compared patient attributes and laboratory data between the welfare and non-welfare groups (Table 1). Median serum 25(OH)D concentration was significantly lower in the welfare group than in the non-welfare group (11.5 [8.7, 14.0] vs. 14.8 [11.2, 19.9] ng/mL, *P* = 0.005). The welfare group exhibited an increased diabetes prevalence compared with the non-welfare group (68.8 vs. 30.0%, *P* = 0.003). There were no significant differences in any other parameters. We observed no significant differences in physical activity levels (IPAQ-SF), serum Alb, or P concentrations between the groups.

### Welfare receipt and risk of VD deficiency

We also examined whether receipt of welfare was a risk factor for VD deficiency. Table 2 shows the results of the single and multiple logistic regression analyses performed using VD deficiency as a dependent variable. This single logistic regression analysis revealed that female sex was a significant risk factor [odds ratio (OR) (95% confidence interval (CI)): 6.04 (2.41, 15.12), *P* < 0.001] and that welfare receipt was not a significant risk factor of VD deficiency [OR (95% CI): 2.75 (0.93, 8.14), *P* = 0.068].

**Table 2:** Single and multiple logistic regression analyses for VD deficiency

	Single regression			Multiple regression (model 1)			Multiple regression (model 2)		
	Odds ratio	*P* value	95% CI	Odds ratio	*P* value	95% CI	Odds ratio	*P* value	95% CI
Age	1.01	0.655	0.97, 1.05	–	–	–	–	–	–
Female sex	6.04	< 0.001^**^	2.41, 15.12	8.17	< 0.001^**^	2.86, 23.36	8.24	< 0.001^**^	2.87, 23.66
ln (BMI)	1.90	0.596	0.18, 20.1	–	–	–	–	–	–
ln (HD vintage)	1.32	0.243	0.83, 2.09	–	–	–	–	–	–
Diabetes	1.62	0.266	0.69, 3.80	–	–	–	–	–	–
History of immunosuppressant use	1.81	0.455	0.38, 8.60	–	–	–	–	–	–
Welfare recipient	2.75	0.068	0.93, 8.14	4.79	0.025^*^	1.22, 18.74	4.41	0.039^*^	1.08, 18.07
IPAQ–SF	1.00	0.966	0.9998, 1.0000	–	–	–	–	–	–
ln (Hb)	2.28	0.803	0.004, 1440.401	–	–	–	–	–	–
ln (CRP)	1.11	0.605	0.74, 1.66	–	–	–	–	–	–
Alb	0.48	0.294	0.12, 1.90	–	–	–	–	–	–
cCa	0.99	0.977	0.54, 1.83	–	–	–	0.88	0.751	0.41, 1.91
P	0.82	0.303	0.55, 1.20	–	–	–	0.88	0.605	0.54, 1.43
ln (iPTH)	0.49	0.020^*^	0.27, 0.90	0.48	0.045^*^	0.24, 0.98	0.48	0.056	0.23, 1.02

Multiple regression analysis (model 1): variables identified as *P* < 0.1 in the single linear regression analysis

Multiple regression analysis (model 2): variables identified as *P* < 0.1 in the single linear regression analysis and serum cCa and P concentrations

Abbreviations: Alb: albumin; BMI, body mass index; cCa, corrected calcium; CI, confidence interval; CRP, C-reactive protein; Hb, hemoglobin; HD, hemodialysis; IPAQ-SF, International Physical Activity Questionnaire-Short Form; iPTH, intact parathyroid hormone; ln, natural logarithm; MET, metabolic equivalent; P, phosphorus

^*^P < 0.05, ^**^P < 0.001

We then conducted a multiple logistic regression analysis (Table 2). In the multiple logistic regression analysis, female sex [OR (95% CI): 8.24 (2.87, 23.66), *P* < 0.001] and welfare receipt [OR (95% CI): 4.41 (1.08, 18.07), *P* = 0.039] were significant risks factors for VD deficiency.

## Discussion

To our knowledge, this is the first study to demonstrate that welfare receipt is a significant risk factor for VD deficiency in patients on maintenance HD. Regardless of whether patients have undergone dialysis, VD deficiency is associated with effects related to frailty and sarcopenia in those with chronic kidney disease (CKD); such effects include low bone density [24], muscle weakness [25], increased fall risk [26], and cognitive impairment [27]. Furthermore, VD deficiency has been linked to infections, including tuberculosis [28], seasonal influenza [29], and novel coronavirus disease 2019 [30], as well as increased mortality [31].

Given the myriad regulatory roles of VD, VD deficiency represents a major global health issue [32–34]. Besides demographic factors, environmental and lifestyle factors influence serum 25(OH)D concentrations. For example, latitude, season, outdoor activity, native VD intake, and body fat composition may be major determinants of serum 25(OH)D concentrations [35]. Guesseous et al. reported that VD deficiency can be observed in CKD patients and in the general population [36]. An observational study has further revealed that VD deficiency worsens as CKD progresses from stage G3 to stage G5D [37].

In general, the causative, risk, and associated factors of VD deficiency in CKD patients (regardless of dialysis status) include age [12], female sex [13, 14], adiposity [15], low physical activity [12], diabetes [14], decreased VD production in the skin [16], calcineurin inhibition [13], decreased hepatic CYP450 due to secondary hyperparathyroidism [17], mineral metabolism abnormality [18, 19], HD vintage [18], anemia [20], and inflammatory state [21]. Nevertheless, maintenance HD rates continue to increase among Japanese patients, especially those with diabetes and/or low physical activity levels [38].

In this study, most patients on maintenance HD were aged ≥ 65 years, and 35.8% had diabetes. However, most patients were not overweight as defined by the World Health Organization (BMI ≥ 25 kg/m^2^) [39]. According to a national survey conducted by the Japanese Society for Dialysis Therapy at the end of 2018, the overall average patient age was 68.75 years, and diabetic nephropathy was the most common primary disease among patients undergoing dialysis (39.0%) [40]. These characteristics are quite similar to those observed among our patients (Table 1). Although the distribution of serum 25(OH)D concentrations within the Japanese population remains unknown, our patients had median serum 25(OH)D concentrations of 14.3 ng/mL, which is close to VD deficiency, defined as serum 25(OH)D concentrations < 12 ng/mL (Table 1) [3].

In this study, patients on welfare accounted for 15.1% of all participants (Table 1)—a rate higher than that previously reported among 7191 Japanese patients undergoing maintenance HD (6.8%) [41]. Serum 25(OH)D concentration was significantly lower in the welfare group than in the non-welfare group (Table 1). Moreover, diabetes prevalence was significantly higher in the welfare group than in the non-welfare group (Table 1).

In Japan, welfare recipients and low-income earners often have a poorly balanced diet, ingesting a large amount of instant noodles and confectioneries, and consuming a small amount of meat, fish, and vegetables [11]. Moreover, the incidence of lifestyle diseases, including diabetes, is higher among welfare recipients than among non-recipients. Despite an increased incidence of health problems, only about 10% of welfare recipients undergo regular health checkups, indicating that they may be less interested in various health-related activities, including dietary and exercise habits [42]. This tendency is possibly similar among patients on maintenance HD receiving welfare, and dietary habits may be responsible for lower 25(OH)D concentrations, given that there were no significant differences in median serum Alb concentrations between the welfare and non-welfare groups. However, there were no significant differences in mean serum P concentrations between the groups (Table 1). There might be differences in food intake containing native VDs that do not significantly affect serum P concentration such as mushrooms. Intake of both native VDs and precursors of 25(OH)D is necessary. VD_3_ is abundant in fish meat and fish liver, while VD_2_ is present in mushrooms. However, low-income earners, whose annual household income is less than approximately 20,000 USD, tend to consume mushrooms less frequently than those whose annual household income is greater than approximately 20,000 USD [43]. Therefore, intake of these foods may be low in welfare recipients undergoing maintenance HD given the tendency for a poorly balanced diet. Such imbalances may also be explained by a significantly higher diabetes prevalence in the welfare group (Table 1). Concurrent increases in the prevalence of type 2 diabetes and hypovitaminosis D have been observed worldwide [44]. Bayani et al. reported that serum 25(OH)D concentrations were significantly lower in diabetes patients than in healthy individuals [45], suggesting that VD insufficiency/deficiency is related to the onset of type 2 diabetes. However, randomized controlled trials have reported controversial results regarding the effect of VD supplementation on glucose metabolism [46, 47].

A positive association exists between physical activity and serum 25(OH)D concentrations [48]; those who exercise outdoors tend to have increased VD concentrations due to sunlight exposure [49]. However, we observed no significant difference in IPAQ-SF scores between the welfare and non-welfare groups (357.0 [0, 2423.0] vs. 823.5 [266.0, 1891.5] MET × minutes/week, *P* = 0.292) (Table 1), possibly because we did not specifically assess the time spent on performing outdoor activities. In fact, in a report from Poland [50], the prevalence of physical activity among the social assistance recipients was much lower than that in the general population. Also, in the report, the two notable reasons why the beneficiaries did not exercise were unwillingness to exercise (25.4%) and poor health condition (12.2%).

The expense for welfare in Japan is approximately 40 billion USD, 75% of which is paid by the state and the remaining 25% by prefectures or municipalities (including special wards). In the USA, Medicare (a program providing insurance for older adults and individuals with disabilities) pays for most treatments for end-stage renal disease (ESRD). According to a 2018 report by the Congressional Research Service, the program spent more than 62,000 USD per patient with ESRD in 2013 [51]. One Italian study also reported that the Lombardy Regional Health Service spent approximately 40,000 EUR per patient with ESRD [52]. Given the large number of patients undergoing HD and receiving welfare, the public health-related economic burden associated with the disease continues to increase. Serum 25(OH)D concentrations, which can predict a potential nutritional status not to be evaluated using serum Alb concentrations, should be appropriately assessed and improved in welfare recipients undergoing HD to prevent the long-term adverse consequences of VD deficiency, including frailty and sarcopenia and control medical costs, based on the statement of the Kidney Disease: Improving Global Outcomes guideline for CKD-MBD in 2009 “The potential risks of vitamin D repletion are minimal, and thus, despite uncertain benefit, the Work Group felt that measurement might be beneficial [53].”

This study had several potential limitations related to its retrospective nature. We could not assess (1) dietary content, (2) nutritional functional food intake, (3) sunlight exposure duration, (4) use of sunshade clothing (tools) and/or sunscreen, (5) skin color, (6) climate-related factors, or (7) occupations of non-welfare recipients that may affect serum 25(OH)D concentrations. Furthermore, the number of patients in each group was relatively small. However, VD supplementation is rare among the Japanese population, since native VD is not available as a pharmaceutical product in Japan [54]. We included patients undergoing HD from two facilities located at different latitudes (Kanagawa and Okinawa). We also used data obtained in early September, a time when serum 25(OH)D concentrations are at their highest throughout the year [55]. Despite these potential limitations, we believe that our study provides substantial insight into the potential for VD deficiency among welfare recipients undergoing maintenance HD.

## Conclusions

This is the first study to demonstrate that welfare receipt is a risk factor for VD deficiency in Japanese patients on maintenance HD, necessitating further studies regarding this relationship. Non-pharmacological regimens involving health-related activities, including well-balanced dietary habits and adequate outdoor activity during the daytime, should be considered to improve health and attenuate medical expenditure, especially in this population.

## Data Availability

The datasets used and/or analyzed in the present study are available from the corresponding author on reasonable request.

## Abbreviations

Alb: Albumin
BMI: Body mass index
Ca: Calcium
cCa: Corrected calcium
CKD: Chronic kidney disease
CKD-MBD: Chronic kidney disease-mineral and bone disorder
COVID-19: Novel coronavirus disease
CRP: C-reactive protein
ESRD: End-stage renal disease
Hb: Hemoglobin
HD: Hemodialysis
IPAQ-SF: International Physical Activity Questionnaire-Short Form
iPTH: Intact parathyroid hormone
IQR: Interquartile range
IRB: Institutional review board
ln: Natural logarithm
MET: Metabolic equivalent
P: Phosphorus
SD: Standard deviation
25(OH)D: 25-Hydroxyvitamin D
VD: Vitamin D
WHO: World Health Organization
